# Ropivacaine-Loaded Poloxamer Binary Hydrogels for Prolonged Regional Anesthesia: Structural Aspects, Biocompatibility, and Pharmacological Evaluation

**DOI:** 10.1155/2021/7300098

**Published:** 2021-09-16

**Authors:** Kelli Cristina Freitas Mariano, Juliana Zampoli Boava Papini, Naially Cardoso de Faria, Daniele Nicoli Cabral Heluany, Ana Luiza Lourençoni Botega, Cíntia Maria Saia Cereda, Eneida de Paula, Giovana Radomille Tófoli, Daniele Ribeiro de Araujo

**Affiliations:** ^1^Human and Natural Sciences Center, Federal University of ABC, Santo André, SP, Brazil; ^2^São Leopoldo Mandic Faculty, São Leopoldo Mandic Research Institute, Campinas, São Paulo, Brazil; ^3^Federal University of São Paulo, Translational Medicine Postgraduate Program, São Paulo, Brazil; ^4^Department of Biochemistry, State University of Campinas, Campinas, São Paulo, Brazil; ^5^Drugs and Bioactives Delivery Systems Research Group–SISLIBIO, Federal University of ABC, Av. dos Estados, 5001 Bl. A, T3, Lab. 503-3. Bangú, Santo André, SP, Brazil

## Abstract

This study reports the development of thermosensitive hydrogels for delivering ropivacaine (RVC), a wide clinically used local anesthetic. For this purpose, poloxamer- (PL-) based hydrogels were synthesized for evaluating the influence of polymer concentration, hydrophilic-lipophilic balances, and binary system formation on biopharmaceutical properties and pharmacological performance. Transition temperatures were shifted, and rheological analysis revealed a viscoelastic behavior with enhanced elastic/viscous modulus relationship (*G*′/*G*^”^ = 1.8 to 22 times), according to hydrogel composition and RVC incorporation. The RVC release from PL407 and PL407/338 systems followed the Higuchi model (*R*^2^ = 0.923–0.989), indicating the drug diffusion from hydrogels to the medium. RVC-PL hydrogels were potentially biocompatible evoking low cytotoxic effects (in fibroblasts and Schwann cells) and mild/moderate inflammation signs on sciatic nerve nearby histological evaluation. *In vivo* pharmacological assays demonstrated that PL407 and PL407/338 evoked differential analgesic effects, by prolonging the sensory blockade duration up to ~340 and 250 min., respectively. All those results highlighted PL407 and PL407/338 as promising new strategies for sustaining analgesic effects during the postoperative period.

## 1. Introduction

The use of biodegradable materials with high biocompatibility has become one of the main objectives in the biomedical sciences. Therefore, the design of innovative systems for drug delivery can offer the modulation of the drug release rate and reduced low toxicity [1]. In special, hydrogels have been widely investigated for biomedical purposes due to their high swelling capability and low toxicity [2, 3].

Hydrogel matrices are structurally organized as polymeric tridimensional networks formed by Van der Waals interactions and hydrogen bonds [4]. Among the main types of polymers used as hydrogel components, poloxamers (PL) have been described as promising systems attributed to their capability for self-assembly in micelles and hydrogels in response to the concentration and physiological temperature. In fact, their composition based on polyethylene oxide-polypropylene oxide-polyethylene oxide- (PEO-PPO-PEO-) linked blocks allows their self-assembly due to hydrophobic block dehydration (PPO), reducing the micellar core and forming a hydrated corona [4, 5]. This phenomenon is followed by hydrogel structural organization as lamellar, hexagonal, and/or cubic phases, modulating the release rate of different drugs, including local anesthetics [6, 7].

Prolonged analgesia is an essential component for effective postoperative pain management. For this purpose, nonsteroidal anti-inflammatory drugs (NSAIDs), analgesic opioids, and local anesthetics, isolated or in association, are used as first-choice pharmacotherapy. However, ineffective duration of analgesia and systemic adverse effects limit their use [8–10]. In this sense, ropivacaine (RVC) was selected as a drug model since it is a widely used amino-amide local anesthetic in regional anesthesia and postoperative pain management due to its lower cardiovascular and neurotoxicity, as well as significant differentiation between sensory and motor blockade duration compared to other local anesthetic agents such as bupivacaine [9, 11, 12].

Local anesthetics in gel formulations had been recently described for topical use [13–15]. However, a variety of systems in hydrogels have been also reported for injectable administration of bupivacaine [16, 17], lidocaine [7, 18–23], and ropivacaine [6, 24]. Additionally, local anesthetic-loaded PL-based hydrogels have been studied as matrices for inorganic compounds such as Ca/Na-rich bioglass [25], prodrug light-triggered release (Zhang et al., 2020), polymeric nanoparticles [26], and lanolin/oleic acid lipid-mixture [27]. All those formulations involve the formation of unique, hybrid, or multiple stimuli-responsive systems, without emphasis on the influence of PL binary matrices on system biopharmaceutical performance. In this sense, it is important to highlight that the use of PL with different polarities or hydrophilic-lipophilic balances (HLB) and molecular weight induces changes on important features attributed to PL-based hydrogels, especially regarding to micellization and gelation processes, mechanical properties, and their therapeutic efficacy [28].

In special, we previously reported the development of PL-based binary hydrogels for prolonged infiltrative local anesthesia. In those studies, the structural analysis evidenced an essential contribution of binary system composition associating their phase organization transitions (from lamellar to hexagonal) and drug release modulation, which were differential for obtaining enhanced sustained drug release [6, 7]. However, both studies were designed for in situ administration, being necessary a more detailed biopharmaceutical evaluation focused on the design of PL binary systems, highlighting differences on hydrophilic-lipophilic balances, polymer final concentration, and molecular weight, as well as their impacts on regional nerve blocks and *in vitro*/*in vivo* biocompatibility. From those analyses, it is possible to predict the system performance for long-duration surgical procedures, regional nerve blockades, and postoperative pain management. All those features highlight the innovative therapeutic strategy described here.

Therefore, we developed RVC-loaded PL407 and PL338 PL-based binary hydrogels, considering PL remarkable differences on physicochemical properties, by evaluating the micellization process thermodynamic parameters, rheological features, drug release mechanisms and, finally, the pharmacological effects and biocompatibility in the sciatic nerve blockade model, looking forward to their application for sustained regional anesthesia and postoperative pain management.

## 2. Materials and Methods

### 2.1. Chemicals and Reagents

Poloxamer 407 (Pluronic® F127) and poloxamer 338 (Pluronic® P108) were purchased from Sigma-Aldrich Chem. Co. (St. Louis. MO, USA). Ropivacaine hydrochloride was a gift from Cristália Ind. Quím. Farm. (Itapira, São Paulo, Brazil). All other reagents were of analytical grade.

### 2.2. High-Performance Liquid Chromatography (HPLC) Analysis Conditions

RVC concentrations were determined by using a HPLC device (Ultimate 3000, Chromeleon 7.2 software, Thermo Fisher Scientific, Waltham, Massachusetts, USA) accoupled to a DAD detector and a gradient pump. The mobile phase was composed of a mixture with acetonitrile and phosphate-buffer solution (60 : 40 *v*/*v*). Analyzes were performed in a C18 column (ODS Hypersil model, Thermo Fisher Scientific, Waltham, Massachusetts, USA, 150 mm × 4.6 mm, 5 *μ*m) at 220 nm, flow rate at 0.5 mL/min, 25°C, and 20 *μ*L of injection. Retention time was 2.4 min. for all samples. Detection (LD) and quantification (LQ) limits were 1.86 and 5.65 *μ*g/mL, respectively, obtained from a previously analyzed standard curve of RVC (5 to 120 *μ*g/mL, *y* = 0.44∗*x* ± 0.072, with a correlation coefficient value of *R*^2^ = 0.9998).

### 2.3. Hydrogels Preparation and Physicochemical Characterization

PL-based formulations were prepared by polymer dispersion in ultrapure water, at 20, 25, and 30% (*m*/*v*) final concentrations for PL407 isolated or in association to PL338 solutions alone or forming binary systems with 5% or 10% of PL338. Polymer's mixtures were maintained at 4°C under magnetic stirring until complete dissolution and transparency. Hydrogel samples were maintained at 4–8°C during all experiments. RVC final concentration was 0.5% for all formulations.

#### 2.3.1. Differential Scanning Calorimetry (DSC) Assays

DSC assays were performed by weighing 20 mg of each hydrogel in hermetic in aluminum pans and analyzing them under three successive cycles as heat-cool-heat mode from 0°C to 50°C, at 5°C/min (DSC 214—Polyma, NETZSCH Instruments, Selb, Germany). An empty aluminum pan was used as blank. Thermograms were presented as heat flux (k·J·mol^−1^) versus temperature (°C), and parameters determined were referred to the micelle formation process considering the beginning (*T*onset), peak (*T*peak), and final (*T*endset) micellization temperatures, as well as enthalpy variation (Δ*H*°) relatively to the micellization peak.

#### 2.3.2. Mechanical Property Analysis: Rheological Studies

Rheological studies were carried out by using an oscillatory rheometer (Kinexus Lab, Malvern Instruments Ltd., Malvern, England, UK) accoupled to a cone-plate geometry (40 mm in diameter). For sol-gel transition temperature determination (*T*sol − gel), aliquots of 1 mL were analyzed under the temperature range of 10–50°C, frequency at 1 Hz, and shear stress of 2 Pa. In a second analysis, elastic modulus (*G*′), viscous modulus (*G*^”^), and viscosity (*η*) parameters were determined by frequency sweep analysis (0.1 to 10 Hz), at shear stress of 2 Pa. and temperature at 37°C. rSpace software for Kinexus® (Malvern Instruments Ltd., Malvern, England, UK) was used for data treatment.

#### 2.3.3. Scanning Electron Microscopy Analysis (SEM)

Hydrogel morphological analyses were performed by SEM. Aliquots of each hydrogel formulation were carefully spread over transparent glass slides and left to dry in a desiccator for 12 h. Dried samples were placed on an aluminum stub and observed on a scanning electron microscope (JEOL 5200LV, JEOL Ltd., Tokyo, Japan) with an accelerating voltage of 5 kV and 400x magnification.

#### 2.3.4. In Vitro Release Assays

For drug release rate determination, a membraneless model was used. A cell glass (donor compartment), filled with 1 mL of each hydrogel formulation, was inserted into a receptor compartment containing 50 mL of 0.01 M of potassium phosphate buffer at pH 7.4, at 37°C, and maintained under stirring (350 rpm). At predetermined intervals (0.5, 1, 2, 4, 8, and 24 h), aliquots (1 mL) from the receptor compartment were withdrawn and analyzed by HPLC. The drug release content was expressed as percentage against time (*n* = 6/formulation).

### 2.4. In Vitro Cytotoxicity and In Vivo Pharmacological Evaluation

#### 2.4.1. In Vitro Cytotoxicity Assays: Sciatic Nerve Schwann Cells and 3T3 Fibroblasts

Cell viability assays were performed by using primary Schwann neuronal cells and *Balb*/*c* fibroblasts (3T3 cells line). Sciatic nerve Schwann cells were obtained from 6 week-old Wistar UNIB rats, and explants were dissected and removed from the epineurium and surrounding neuronal tissues under aseptic conditions, as previously described by Renó et al., 2019.The protocol was approved by the State University of Campinas Institutional Animal Care and Use Committee, following the recommendation from the Guide for the Care and Use of Laboratory Animals (protocol # 4033-1). Cells were maintained in humid atmosphere (95% O_2_ and 5% CO_2_) at 37°C, while they reached 80% of confluence, and culture medium was periodically changed (every 48–72 h). Cell neuronal isolation was confirmed by epifluorescence antibodies, anti-vimentin (1 : 300; Dako, Glostrup, Denmark), anti-S100 (1 : 300; Dako, Glostrup, Denmark), and anti-AE1/AE3 (1 : 75; Dako, Glostrup, Denmark) for staining cytoskeleton, neuronal, and epithelial origin, respectively. The positive immunostaining for vimentin and S100 confirmed the neuronal cell origin.

For cytotoxicity studies, 3T3 fibroblasts and Schwann cells were seeded in 96-well plates (2.10^4^ cells/well) in culture medium DMEM supplemented with 10% fetal bovine serum at 37°C and humified atmosphere, for 24 h. Cells were treated for 4 h with different RVC concentrations (0.0625 to 1.25 mg/mL). Hydrogels were evaluated by using indirect test according to ISO 10993-5 (2009). Cell viability percentage was determined by MTT reduction test.

For IC_50_ calculation, concentration absolute values were converted into logarithms and cell viability percentages were normalized according to those values from negative control. From these data, a dose response inhibition curve was obtained for each treatment and IC_50_ was determined by interpolating the *x*-axis, from a nonlinear regression analysis, as previously described by [29].

#### 2.4.2. Animals

Fifty-four male Wistar adult rats, weighing from 300 to 350 g, were obtained from São Leopoldo Mandic Faculty (Biotério Central-SLMandic). Animals were maintained under 12 h light/dark cycles, with water and food *ad libitum*, room temperature at 22 ± 3°C. The protocol was approved by São Leopoldo Mandic Faculty Institutional Animal Care and Use Committee, with recommendations from the Guide for the Care and Use of Laboratory Animals (protocol # 2018/035).

#### 2.4.3. Sciatic Nerve Blockade: Motor and Sensory Function Evaluation

Sciatic nerve blockade model was used to assess the regional local anesthetic effects. Animals were treated by formulation injection (0.4 mL) into the greater trochanter region of the right posterior limb, in the region close to the sciatic nerve [30]. RVC concentration was 0.5% for all formulations. Experimental groups (*n* = 6/group) were assigned as follows: G0-RVC, G1-PL407 (30%) + RVC, G2-PL407 (25%) + RVC, G3-PL407/338 (25/5%) + RVC, G4-PL407/338 (20/10%) + RVC, G5-PL407 (30%), G6-PL407 (25%), G7-PL407/338 (25/5%), and G8-PL407/338 (20/10%).

The induction of motor block was verified by the loss of motor control of the right posterior limb (anesthetized side) and its intensity evaluated by using score values: 0 (normal use of the injected limb), 1 (inability to flex the limb completely), and 2 (total inability of using the limb) [31]. The evaluation period was limited from the first 5 minutes every minute after formulation injection and, subsequently, at intervals of 5 to 10 minutes until the total recovery of movements of the anesthetized limb of the animal was verified (at least 1 hour of observation). For sensory blockade assessment, pain withdrawal threshold to pressure (PWTP) was detected by animal response (paw removing) to a mechanical stimulus on the hind paw [32]. For this purpose, an analgesimeter (Ugo Basile, Varese, Italy) device was used, which applies a linearly increased force (in grams) by a plastic end on the injected paw. Fifteen minutes after injection, the animal's paw was placed under the plastic end and a pressure was applied and constantly increased until the animal removed his paw, as a sign of nociception. Analgesia was defined by the pressure increase at least 50% greater than that observed for control groups (drug-free treatment). The analgesia end point was established when treated animals presented a similar pressure response (PWTP, in g) to control groups. A maximum value (cut-off) of 350 grams was limited for avoiding tissue injuries and excessive stimulation of nociceptors.

#### 2.4.4. Biocompatibility: Tissue Morphological Evaluation

After motor and sensory blockade evaluation, animals were euthanized under general anesthesia (urethane 1 g/kg and *α*-chloralose 50 mg/kg) after 2 or 7 days, to evaluate the tissue morphological damage (*n* = 3/each evaluation time). The sciatic nerve adjacent tissue was excised, and cross-sections (5 *μ*m thick, 40 *μ*m deep) were prepared by staining with hematoxylin and eosin and qualitatively analyzed by a blinded examinator using the following score system to determine local inflammation and leukocyte infiltration: (1) <25% total area without infiltrate, injury, or necrosis (mild inflammation), (2) 25%–50% area with inflammatory infiltrate, injury, or necrosis (moderate inflammation), and (3) >50% area with injury and displays necrosis areas (severe inflammation) [30].

### 2.5. Statistical Analysis

Data were expressed as mean ± standard deviation (SD) and analyzed by software GraphPad Prism version 6.0 (GraphPad Software, San Diego, CA, USA). For statistical comparisons, one-way analysis of variance (one-way ANOVA) and the post hoc Turkey-Kramer test were applied, considering *p* < 0.05 as statistical difference.

## 3. Results and Discussion

### 3.1. Hydrogel Physicochemical and Morphological Analysis

#### 3.1.1. DSC Analysis: The Micellization Process Is Modulated by Drug Incorporation and Binary System Formation

Table 1 displays thermodynamic parameters relative to the micellization process, *T*onset, *T*peak, *T*endset, and enthalpy (Δ*H*°), for different hydrogel formulations. In general, results obtained were dependent on PL final concentration and the PL407/PL338 ratio. All formulations were responsive to the heat-cool-heat cycle, indicating that hydrogel thermoresponsive features were preserved even after RVC incorporation.

For all formulations, transition temperatures revealed an endothermic peak around 19°C, for 20 and 25% PL407, and at ~16°C for 30% PL-systems (PL407 and PL407/338), corresponding to the micellization temperature. Those differences on *T*peak values can be attributed to the increase on PL final concentration, even considering binary systems. PL-based systems tend to self-assemble in response to temperature changes, due to PPO central block dehydration [33], dependent on the polymer final concentration and HLB values for their binary systems. Hence, the micellization process is an intermediate step during PL-hydrogel formation and factors that affect the micellization are also able to change their thermogelation (Perinelli et al., 2014).

The formulations composed of PL407 and PL407/338 showed similar Δ*H*° values, with a slight decrease after RVC incorporation being observed. For PL binary systems, self-assembly is necessary to consider the differences on HLB values for PL407 (22) and PL338 (28), as a consequence of their PEO : PPO relationships, being 3 : 1 and 5 : 1 for PL407 and PL338, respectively. The formation of more hydrated systems composed of a PL-binary mixture can allow the complete drug dispersion, since RVC was incorporated as salt form, even it is a relatively hydrophobic drug (partition coefficient_liposome/water_ = 132, [31]), explaining the differences on temperatures and Δ*H*° values. Therefore, the incorporation of drugs [5, 6, 34] and other additives, such as proteins (Perinelli et al., 2014), natural polymers [7], and oil phases [15, 27, 35], can disturb this process, which is detected by *T*onset, *T*peak, *T*endset, and Δ*H*° displacements.

#### 3.1.2. Rheological Studies: Hydrogel Structural Organization

Hydrogel formulations were analyzed under a temperature range and frequency sweep for determining their mechanical properties, such as elastic (*G*′) and viscous (*G*^”^) moduli, viscosity (*η*∗), and sol-gel transition temperature (*T*sol − gel). As also shown by DSC analysis, all hydrogels displayed a thermosensitive and viscoelastic behavior, indicating the compatibility among their components and the preservation of this feature under physiological conditions. All results are presented on Table 2.

Rheograms for hydrogels composed of PL407 as unique or as binary systems with PL338 showed a viscoelastic behavior, where *G*′ values predominated over *G*^”^ by ca. ~1.8 to 22 times, according to PL final concentration, in association with PL338 and drug incorporation. At 20 and 25% PL407 concentrations, RVC hydrogels presented lower *G*′/*G*^”^ relationships than that observed for 30% polymeric final concentrations (Table 2).

In addition, frequency sweep analysis revealed that *G*′ > *G*^”^ for all formulations, predicting the systems viscoelasticity preservation at physiological temperature.

The highest values of *G*′/*G*^”^ relationships were obtained for 30% PL407 systems, while the incorporation of a hydrophilic copolymer, PL338, and RVC reduced *G*′/*G*^”^, indicating the influence of both components on hydrogel structural organization. On the other hand, the viscosity (*η*∗) parameter was also changed in response to different hydrogel compositions, with higher values for binary systems being observed when compared to PL407 unique hydrogels. The RVC effects were mainly related to increased viscosity in formulations with high PL407 concentrations.

Most of PL-based hydrogels formulations were thixotropic materials, an important feature for studying fluids. This feature demonstrates the ability of a gel to liquefy when a certain amount of heat or mechanical force are applied, such as shear or vibrations. After those stimulus applications, the materials return to its original structure. This is an essential feature for predicting the hydrogel injectability, being fluid at low temperatures and assuming the hard-gel structure at the body temperature, maintaining the local anesthetic formulation at the application site. Those observations are also in agreement with previous reports in the literature [6, 7, 22].

#### 3.1.3. Morphological Analysis by Scanning Electron Microscopy (SEM)

**Figure 1:**
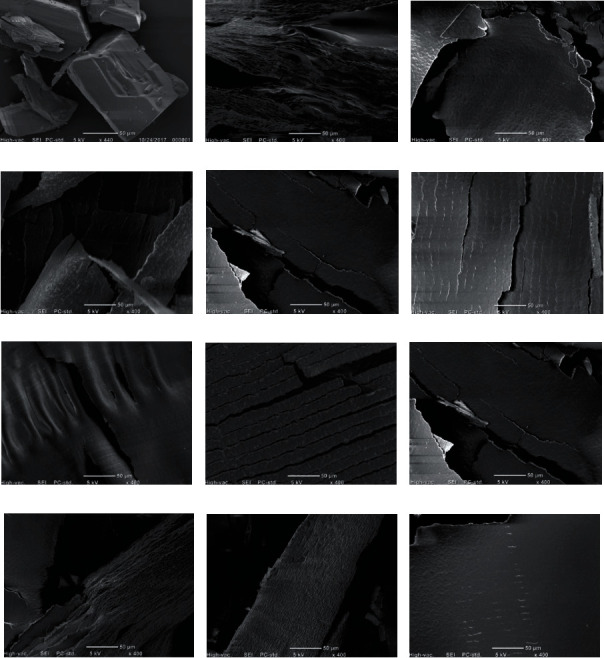
Scanning electron microscopy obtained for RVC (a), PL338 (b), PL407(20) (c), PL 407(20)-RVC (d), PL407(25) (e), PL 407(25)-RVC (f), PL 407(30) (g), PL407(30)-RVC (h), PL407/338(25 : 5) (i), PL407/338(25 : 5)-RVC (j), PL407/338(20/10) (k), and PL407/338(20/10)-RVC (l).

SEM analysis showed the typical morphology of RVC crystals with a rectangular shape. In a different way, PL407 samples were observed as layered blocks, while PL407/338 binary systems presented structures with porous morphology. All samples were homogenous and RVC crystals were not observed, possibly indicating the complete drug dispersion into the hydrogels, in agreement with previously described morphologies for PL-based systems (Figure 1) [6, 27].

### 3.2. Release Assays and Mechanisms

**Figure 2:**
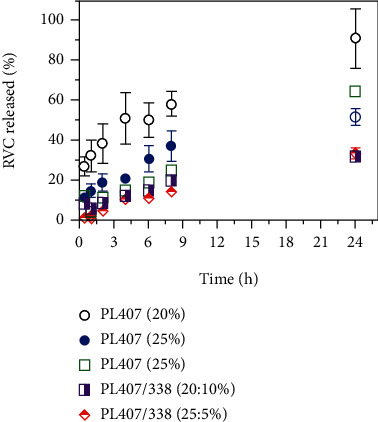
RVC release profiles for unique PL407 and binary PL407/338 systems (*n* = 6/formulation).

Figure 2 displays RVC release profiles from PL407 and PL407/338 hydrogel formulations. In general, drug release was gradual and reached the highest percentage values at 24 h. For 20, 25, and 30% PL407 systems, RVC released was progressive reaching ~90, 50, and 60%, respectively. However, binary systems reduced the drug release against time, showing a sustained profile with ~30% of drug percentages into the receptor medium, for both 20–10% and 25–5% PL407/338.

In order to determine the release constants (Krel) and their mechanisms, data were analyzed by using the following mathematical models: zero order (equation (1)), Higuchi (equation (2)), and Korsmeyer-Peppas (equation (3), as expressed by the following equations:

(1) $$\begin{array}{c} Q_{t}=Q_{0}+K_{0}^{t}, \end{array}$$

where *Q*_*t*_ is the amount of drug released against time *t*, *Q*_0_ is the initial drug concentration, and *K*_0_ the zero-order release constant.

(2) $$\begin{array}{c} Q_{t}=K_{H}t^{1/2}. \end{array}$$

*K*_*H*_ is the release constant determined according to Fick's law, and *Q*_*t*_ is the drug concentration released, and

(3) $$\begin{array}{c} \frac{M_{t}}{M_{\infty }}=K_{kP}t^{n}. \end{array}$$

*M*_*t*_/*M*_∞_ is the drug-released fraction as a function of time *t*, *K*_*kP*_ is the release constant, and *n* is the release exponent. Release mechanisms are determined according to the *n* value of 0.45 (Fickian diffusion), 0.45 < *n* < 0.89 (non-Fickian diffusion or anomalous transport), and *n* > 0.89 (super case II transport).

Comparisons among formulations revealed the influence of PL final concentration on Krel values for unique systems, since high PL407 concentrations determined a sustained RVC release profile (Table 3). For binary systems, Krel values were slightly lower compared to those observed for PL407, highlighting that the formation of a more hydrated system, due to PL338 incorporation, was also able to modulate the drug release rate. In fact, DSC and rheological analysis showed the influence of RVC on hydrogel structural organization improvement, especially for PL407/PL338, explaining the sustained release profile. Considering *in vitro* release profiles, PL final concentrations of 25 and 30%, as PL407 or PL407/338, were selected for *in vivo* studies.

Additionally, the mathematical modelling analysis indicated that RVC release followed the Higuchi model and, hence, the Fick's Law, which is modulated by the drug diffusion process from hydrogels until the receptor medium. All this process can be also associated to hydrogel dissolution and polymeric chain relaxation, due to the PL high water solubility and the fact that experiments were performed by using a membraneless model. Other important point is that in case of local administration, there is a contact between interstitial fluid and hydrogels surface, where the dissolution process and drug diffusion take place until the drug reach the site of action.

On the other hand, PL407 (30%) followed the zero-order model, which can be possibly explained by the formation of a network with small mesh size among intermicellar spaces due to the high PL concentration. The other important point is that the incorporation of RVC as a salt form tends to induce micelle packing, enhancing the PPO micellar core dehydration and promoting the formation of a more intricated crystalline internal structure. Taken together, both factors were essential for shifting the release mechanism preferentially to surface erosion and degradation. In fact, reports in the literature discuss the influence of different hydrogel compositions on the zero-order release mechanism considering the mesh network properties, after drug incorporation or polymer crosslink and the hydration effects on polymeric chain relaxation [36]. In fact, previous studies also showed zero-order mechanisms driven by surface processes, such as a copolymer composed of poloxamer-L-lactic acid-formed hydrogels with gradual erosion kinetics [37], and high polymeric concentrations (35%) of PEG polymers crosslinked to *ß*-cyclodextrins or cholesterol were able to produce an inner structure with small spaces on a mesh size, which shifted the release from Fickian diffusion to zero-order mechanisms [38]. In other reports with distinct delivery system compositions, such as lidocaine/prilocaine chitosan-pectin-blended nanofilms, Fickian diffusion was also the main mechanism for explaining the drug release [39]. Similar results were reported by Rasool et al., 2020, since lidocaine release from carrageenan-alginate hydrogels was also driven by diffusion mechanisms, indicating that the drug is the main entity that moves from the formulation towards the dissolution medium.

### 3.3. In Vitro Cytotoxicity, Pharmacological, and Histological Evaluation: RVC-Loaded Hydrogel Is Potentially Biocompatible and Prolonged Sensory Blockade Duration

**Figure 3:**
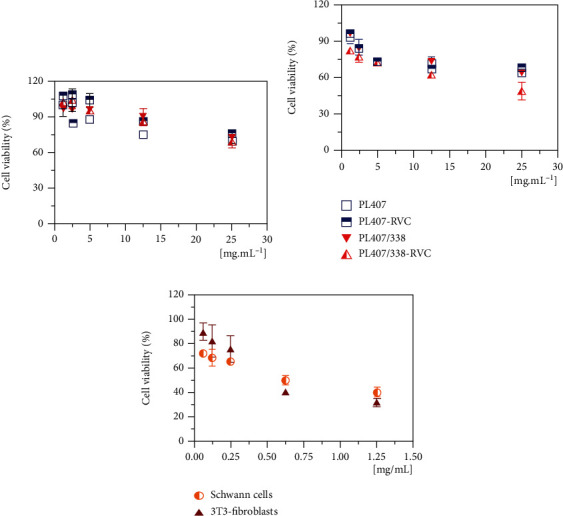
Cell viability percentages after Schwann (a) and 3T3-fibroblast (b) cell treatment with PL-hydrogel formulations and plain RVC (c) evaluated by MTT reduction test.

Cell viability assays were performed in both 3T3-fibroblasts and neuronal Schwann cells for comparing formulations of cytotoxic effects, as shown in Figures 3(a) and 3(b). Treatment with RVC evoked cytotoxic effects on both cell lines, with viability percentages of ~40 and 32% (at 1.25 mg/mL RVC), for neuronal and fibroblasts, respectively (Figure 3(c)), as also pointed out by differences on IC_50_ values for both cells, fibroblasts (0.35 ± 0.05 mg/mL), and neuronal (0.42 ± 0.04 mg/mL).

On the contrary, cell treatment with PL-based formulations induced higher viability percentages when compared to plain RVC. Comparisons among formulations revealed similar cytotoxic effects for PL407 and PL407/338 in Schwann cells (68% of cell viability) with IC_50_ values of 5.1 ± 0.4 mg/mL and 13.5 ± 0.5 mg/mL, respectively. After RVC incorporation, IC_50_ values were higher compared to plain RVC (*p* < 0.001), indicating that incorporation into hydrogels reduced the cytotoxicity evoked by drug. This is an essential factor considering that Schwann cells are adjacent to neurons and can be tightly in contact with the local anesthetic site of application. Schwann cells have been used as models for local anesthetic cytotoxicity evaluation, especially comparing bupivacaine and ropivacaine, showing a time- and concentration-dependent effect (Yang et al., 2011). Then, the use of drug delivery systems is an interesting strategy for developing new and biocompatible pharmaceutical formulations, avoiding injuries to the nerves and spinal cord. In this sense, recent studies report the effects of nanomaterials in Schwann cell cultures looking forward their use for peripheral nerve regeneration by using graphene mesh supported in alginate hydrogels [40] and polymeric nanoparticles [41] as well as ethosome-gels for peripheral neuropathy treatment [42].

Differences among formulations were also observed after 3T3 treatment, since PL407/338 reduced the cell viability to 49.9%, which can be possibly attributed to the drug availability enhancement to the medium. However, incorporation of RVC into the hydrogels significantly increased (*p* < 0.001) the IC_50_ values compared to plain RVC (0.35 ± 0.05 mg/mL), being 2.6 ± 0.36 and 3.9 ± 0.19 mg/mL, for PL407 and PL407/338, respectively. Those results agree with other findings, where RVC-loaded PL-based hydrogel cytotoxicity was assessed in 3T3 cells at similar concentrations [6]. Additionally, PL-based formulations have been reported to induce low cytotoxic effects in other cell types such as V79, hepatocytes (Santos et al., 2015), and Vero cells [7]. In other reports, cytotoxicity evoked by different types of hydrogels was also assessed in 3T3 cells, such as lidocaine-loaded nanostructured lipid carriers in chitosan-pectin matrices for mucosal application [39]; lidocaine-loaded carrageenan-alginate hydrogels (Rasool et al., 2020) and ropivacaine/dexmedetomidine coloaded nanostructured lipid carriers in hyaluronic acid hydrogels (Yang et al., 2019), showing promising results for reducing drug cytotoxicity.

**Figure 4:**
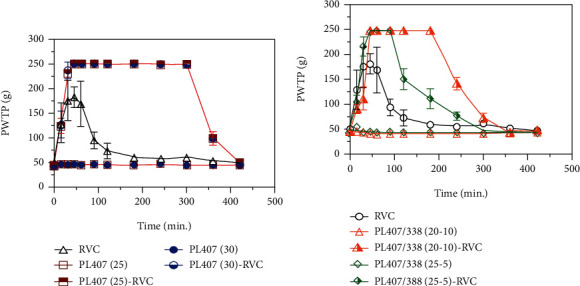
*In vivo* sensory function evaluation after sciatic nerve blockade in rats treated with plain RVC (a, b); unique PL407 (a) and binary PL407/338 hydrogels (b). Data expressed as mean ± SD (*n* = 6 animals/formulation).

In order to investigate the *in vivo* performance of RVC-loaded hydrogels, the sciatic nerve blockade model provided information about motor and sensory block functions. The pain withdrawal threshold to pressure (PWTP) assays indicate the animal response to nociceptive effects. Additionally, the total local anesthetic effect was estimated by using the area under the effect-time curve (AUEC) and the duration of sensory blockade was also evaluated, as depicted on Figure 4 and Table 4.

After sciatic nerve blockade, hydrogels did not induce motor blockade, different from results obtained for RVC solution (motor blockade intensity scores from 2 to 3). This fact can be explained by the prolonged RVC latency due to its incorporation into the formulations and low release from hydrogels to the surrounding tissues, corroborating with *in vitro* release assays. As also reported by previous studies, RVC is characterized by its capability to induce significant separation between motor and sensory blockade, which was enhanced after its loading in PL hydrogels. In fact, as reported by literature, sustained release local anesthetic formulations tend to block sensory rather than motor pathways, being one of the main features for sustaining drug release and regional nerve blockade from the surgical to the postoperative period [24, 31, 43].

The evaluation of sensory blockade showed that RVC induced a duration of analgesia until 100 min after injection, while hydrogel formulations significantly (*p* < 0.001) prolonged this effect up to 340 ± 30 min for 25 and 30% PL407 and 220 ± 30 and 260 ± 33 min for 25/5 and 20/10% PL407/338, respectively (Figure 4). Hence, AUEC values followed similar profiles, where the highest values were obtained after treatment with 25 and 30% PL407 formulations (AUEC_0−420_ = 86154 ± 635.3 and 86323 ± 687.6, respectively) providing a significantly (*p* < 0.001) sustained analgesic effect when compared with plain RVC and binary systems. All statistical comparisons are presented on Table 4.

**Figure 5:**
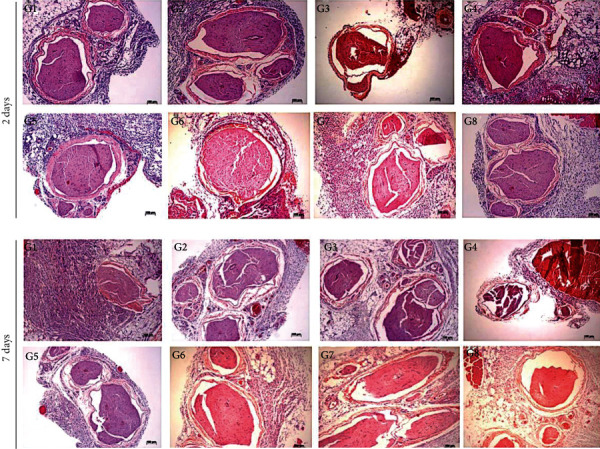
Sciatic nerve adjacent tissue morphological evaluation 2 and 7 days after animal treatment with PL407 and PL407/338 hydrogels. Scale bar: 100 *μ*m. G1: PL407 (30%) + RVC, G2: PL407 (25%) + RVC, G3: PL407/338 (25/5%) + RVC, G4: PL407/338 (20/10%) + RVC, G5: PL407 (30%), G6: PL407 (25%), G7: PL407/338 (25/5%), and G8: PL407/338 (20/10%) (*n* = 6/formulation.

In order to evaluate the local toxic effects, sciatic nerve adjacent tissues were removed and evaluated by a score systems for inflammatory infiltration and necrosis at two different times after injection (2 and 7 days). As observed on Figure 5, all hydrogel formulations did not induce pronounced local tissue toxicity, with score values from mild (1) to moderate (2) for both periods of evaluation being observed (Table 4), indicating the potential biocompatibility of hydrogel systems.

Different reports in the literature described the development of RVC delivery systems due to its interesting clinical features such as lower cardio- and neurotoxicity, compared to other agents like bupivacaine. One of the most recently used RVC delivery strategies include stimuli-sensitive hydrogels, such as PL407/hyaluronate [24] and PL407/188 binary systems [6]. However, those systems were designed for infiltrative local anesthesia by the subcutaneous route. Herein, we proposed the use of RVC-loaded PL-based systems for regional nerve blockade providing us detailed evaluation of their biopharmaceutical performance. Despite that the latency of local anesthesia was not reduced, it was possible to highlight some important findings: (i) PL-based hydrogels significantly prolonged the sensory without evoking motor blockade enhancement, allowing motor function recovery but sustaining analgesic effects during the postoperative stage and (ii) the improvement on regional blockade is determined by both PL final concentration and composition, since the association of PL407/338 (20/10 and 25/5%) evoked less-pronounced analgesic duration when compared to 30% PL407. On the contrary, other RVC-loaded binary systems composed of PL407/188 were effective for prolonging infiltrative analgesia [6], indicating that the incorporation of more hydrophilic and distinct molecular weight compared to PL407, such as PL188 (HLB = 29, MW = 86400) or PL108 (HLB = 28, MW = 14600) exerts differential effects according to the extension, procedure complexity, and area of nerve blockade. This feature is inherent to formulation dispersion throughout adjacent tissues and their capability for maintaining the drug contact on nerves nearby.

## 4. Conclusions

This study reported the design and pharmacological evaluation of RVC-loaded PL-based hydrogels as unique PL407 and PL407/338 binary systems looking forward to applications for regional anesthesia. Physicochemical studies revealed the RVC influence on hydrogel structural organization, as evidenced by changes on thermodynamic and mechanical properties. Additionally, RVC-loaded hydrogels exhibit a viscoelastic behavior which is essential for injectable formulations. PL final concentrations and the formation of binary systems modulated the drug release rate, following a Fickian diffusion model from hydrogels to the medium. Cytotoxic and histological evaluation showed the potential hydrogel biocompatibility associated to prolonged analgesic effects without inducing motor blockade, essential features for sustaining analgesia until the postoperative period.

## Figures and Tables

**Table 1:** Micellization onset (*T*onset), peak (*T*peak), endset (*T*endset), and enthalpy variation (Δ*H*°) values for PL-based hydrogels.

PL (%)	Formulations	*T*onset (°C)	*T*m (°C)	*T*endset (°C)	Δ*H*° (J/g)
20	PL407	15.2 ± 0.50	18.5 ± 0.54	23.7 ± 0.37	2.1 ± 0.04
	PL407-RVC	15.5 ± 0.47	18.7 ± 0.49	23.3 ± 0.22	1.9 ± 0.09
25	PL407	15.0 ± 0.48	18.6 ± 0.43	24.5 ± 0.90	2.5 ± 0.18
	PL407-RVC	13.7 ± 0.04	17.8 ± 0.65	22.8 ± 1.60	2.4 ± 0.19
30	PL407	12.3 ± 0.41	15.7 ± 0.10	20.7 ± 0.31	2.9 ± 0.06
	PL407-RVC	11.7 ± 0.21	15.4 ± 0.16	21.4 ± 0.95	2.6 ± 0.11
20-10	PL407/338	12.1 ± 0.30	16.0 ± 0.15	21.2 ± 0.10	2.6 ± 0.11
	PL407/338-RVC	12.3 ± 0.50	16.2 ± 0.38	21.8 ± 0.78	2.1 ± 0.04
25-5	PL407/338	11.7 ± 0.37	16.8 ± 0.44	22.6 ± 0.19	2.3 ± 0.18
	PL407/338-RVC	11.9 ± 0.65	16.9 ± 0.46	22.3 ± 0.60	1.9 ± 0.05

**Table 2:** Elastic (G′) and viscous (G^”^) moduli, viscosity (mPa·s) at 37°C and sol-gel transition temperatures (*T*sol − gel, °C) for PL 407 and PL338 hydrogel formulations.

PL (%)	Formulations	G′ (.10^5^ mPa at *T*sol − gel)	G^”^ (.10^5^ mPa at *T*sol − gel)	G′/G^”^ (at *T*sol − gel)	*η*∗ (.10^5^ mPa·s at *T*sol − gel)	*T*sol − gel (°C)
20	PL407	246.1	135.2	1.82	1044	32.5
	PL407-RVC	295.3	78.4	3.76	2334	30.7
25	PL407	287.5	66.6	4.31	1658	24.5
	PL407-RVC	426.7	87.7	4.86	2473	24.1
30	PL407	1007	45.6	22.0	2218	21.4
	PL407-RVC	1116	65.0	17.2	2528	21.9
20-10	PL407/338	778.1	82.6	9.40	2204	23.8
	PL407/338-RVC	999.7	78.6	12.7	2587	23.8
25-5	PL407/338	876.5	63.6	13.8	2144	23.4
	PL407/338-RVC	638.4	48.9	13.0	2605	23.1

**Table 3:** RVC release constants (Krel) and mathematical model parameters for PL407 and PL407/338 hydrogels.

Formulations (PL, %)	Zero order		Higuchi		Korsmeyer-Peppas		
	Krel (%.h^−1^)	*R* ^2^	Krel (%.*h*^−1/2^)	*R* ^2^	Krel (%.*h*^−*n*^)	*R* ^2^	*n*
PL407 (20)	2.5	0.940	15.0	0.989	30.3	0.981	0.31
PL407 (25)	1.6	0.880	10.0	0.967	13.4	0.979	0.30
PL407 (30)	2.0	0.974	11.3	0.923	7.7	0.704	0.52
PL407/338 (20-10)	1.1	0.947	6.2	0.973	7.1	0.910	0.45
PL407/338 (25-5)	1.3	0.978	7.8	0.984	1.8	0.964	1.00

**Table 4:** Analgesia duration, area under the effect-time curve (AUEC) and histological scores after RVC-loaded PL-based hydrogels.

Groups	Formulations (PL, %)	Mean time analgesia duration (min.)	AUEC_0-420_ (min.)	Histological score median (minimum-maximum limits)	
				2 days	*7* days
Control (drug-free hydrogels)	PL407 (25)	—	—	2 (2–3)	2 (2–3)
	PL407 (30)	—	—	2 (1–3)	2 (1–3)
	PL407/338 (20/10)	—	—	2 (2–3)	2 (2–3)
	PL407/338 (25/5)	—	—	3 (2–3)	2 (2–3)
Test (RVC-loaded hydrogels)	Plain RVC	100 ± 25^a,b,c,d∗∗∗^	32814 ± 1294	2 (2–3)	2 (2–3)
	PL407 (25)-RVC	340 ± 31^e,f∗∗∗^	86154 ± 635.3	2 (2–3)	2 (2–3)
	PL407 (30)-RVC	340 ± 32^g∗∗∗,h∗∗^	86323 ± 687.6	2 (1–3)	2 (1–3)
	PL407/338 (20/10)-RVC	220 ± 31^c∗∗∗^	63528 ± 754.7	2 (2–3)	2 (2–3)
	PL407/338 (25/5)-RVC	260 ± 33	47106 ± 1244	2 (1–3)	2 (1–3)

^∗^Data expressed as median ± SD (n = 6/formulation) (ANOVA/Tukey-Kramer): ^∗∗∗^*p* < 0.001, ^∗∗^*p* < 0.01, ^∗^*p* < 0.05. ^a^RVC vs PL407(25)-RVC; ^b^RVC vs PL407 (30)-RVC; ^c^RVC vs PL407/338 (25/5)-RVC; ^d^RVC vs PL407/338(20/10)-RVC; ^e^PL407 (25)-RVC vs PL407/338(25/5)-RVC; ^f^PL407(25)-RVC vs PL407/338(20/10); ^g^PL407(30)-RVC vs PL407/338(25/5)-RVC; ^h^PL407(30) vs PL407/338(20/10).

## Data Availability

All data will be available upon author request.

## Supplementary Materials

Supplementary Materials Ropivacaine-loaded hydrogels: composition, rheological analysis, in vivo biocompatibility, and pharmacological efficacy evaluated by sciatic nerve blockade.
