# Supplementary material for: Ropivacaine-Loaded Poloxamer Binary Hydrogels for Prolonged Regional Anesthesia: Structural Aspects, Biocompatibility, and Pharmacological Evaluation
Source: Biomed Res Int. 2021 Sep 16;2021:7300098. doi: 10.1155/2021/7300098 (PMC8460376; doi:10.1155/2021/7300098)
Supplement: Supplementary Materials — Ropivacaine-loaded hydrogels: composition, rheological analysis, in vivo biocompatibility, and pharmacological efficacy evaluated by sciatic nerve blockade. [file 7300098.f1.pdf]

SUPPLEMENTARY MATERIAL – Ropivacaine loaded hydrogels: composition, rheological analysis, in vivo biocompatibility and pharmacological efficacy evaluated by sciatic nerve blockade

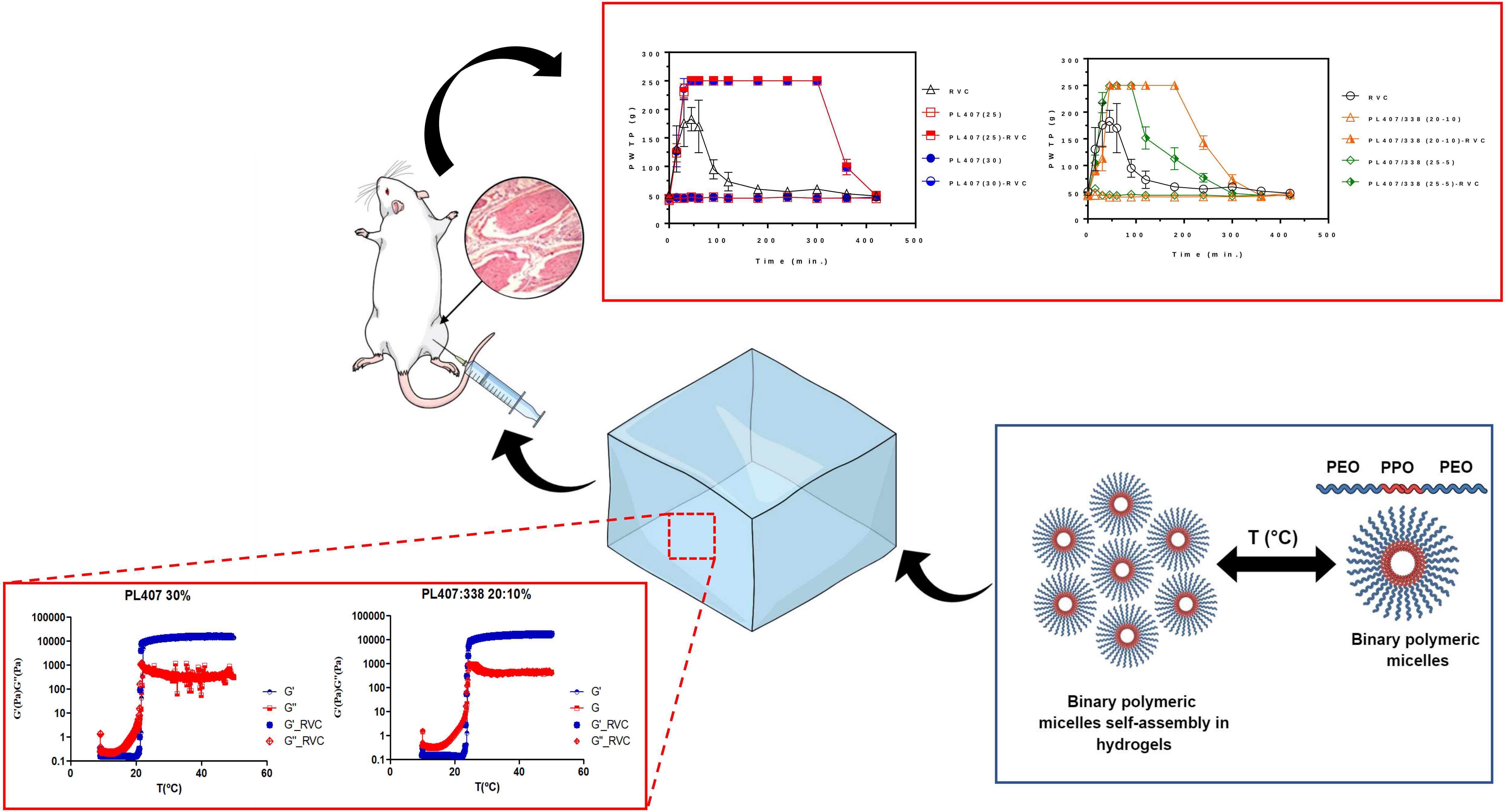
PWTP (g)
Time (min.)
RVC
PL407(25)
PL407(25)-RVC
PL407(30)
PL407(30)-RVC
PL407/338 (20-10)
PL407/338 (20-10)-RVC
PL407/338 (25-5)
PL407/338 (25-5)-RVC
PL407 30%
PL407:338 20:10%
G'(Pa)G"(Pa)
T(°C)
G'
G"
G'_RVC
G"_RVC
G
PEO
PPO
PEO
T (°C)
Binary polymeric micelles self-assembly in hydrogels
Binary polymeric micelles
